# Application of the latest CLSI EP guidelines in light-initiated chemiluminescent assay for testosterone

**DOI:** 10.5937/jomb0-58894

**Published:** 2026-03-17

**Authors:** Ai Liang, Cao Xiao-Jing, Ren Xin-Xin, Kang Lan, Zhang Dan, Xin Chang

**Affiliations:** 1 Women & Infants Hospital of Zhengzhou, Department of Laboratory Science, Zhengzhou, China; 2 The Second Medical Center of Chinese PLA General Hospital, National Clinical Research Center for Geriatric Diseases, Beijing, China; 3 Chemclin Diagnostics Co., Ltd. Standardisation & Performance Evaluation Laboratory, Beijing, China

**Keywords:** testosterone, light-initiated chemiluminescent assay, CLSI, evaluation, verification, testosteron, svetlom aktiviran hemiluminiscentni test, CLSI, evaluacija, verifikacija

## Abstract

**Background:**

A commercial light-initiated chemiluminescent assay (LiCA) for testosterone (T) measurement was developed. This study comprehensively evaluated the analytical performance of LiCA-T following the Clinical and Laboratory Standards Institute (CLSI) Evaluation Protocols (EP) series guidelines.

**Methods:**

From July 1st 2023, both the manufacturer and clinical end-users conducted rigorous performance evaluations in accordance with CLSI EP guidelines. Assessment included precision, linearity, accuracy/trueness, detection limits, and interference testing using collected clinical samples. All the tests were carried out using LiCA® 800 (Chemclin, Beijing, China), and the reagent of lot 2202.

**Results:**

Manufacturer evaluations demonstrated excellent assay performance: repeatability (1.41-4.39%), intermediate imprecision (1.62-7.29%), and reproducibility (1.62-7.22%). The method showed linearity across 0.10-16.64 ng/mL (ADL=10%). Method comparison with MS reference yielded a limit of agreement (LoA) of -19.5% to 14.4%, with ≤10.00% bias at medical decision points. Sensitivity parameters included: LoB (0.041 ng/mL), LoD (0.049 ng/mL), and LoQ (0.060 ng/mL at 20% imprecision). The assay demonstrated robust interference resistance against haemoglobin (2 mg/mL), biotin (20 ng/mL), triglyceride (3 mg/mL), and bilirubin (0.157 mg/mL). All manufacturer claims were successfully verified by independent clinical end-user testing.

**Conclusions:**

The LiCA-T assay satisfies all performance criteria specified in CLSI latest EP guidelines (including EP06-Ed2 and EP07-Ed3), demonstrating reliable analytical performance across all critical validation parameters for clinical testosterone measurement.

## Abstract (sr)

**Uvod:**

Razvijen je komercijalni svetlom aktiviran hemiluminiscentni test (LiCA) za kvantitativno određivanje testosterona (T). Cilj ove studije bio je sveobuhvatno ispitivanje analitičkih performansi LiCA-T u skladu sa smernicama serije protokola za evaluaciju (EP) Clinical and Laboratory Standards Institute (CLSI).

**Metode:**

Od 1. jula 2023. godine, i proizvođač i klinički korisnici su sproveli detaljne evaluacije performansi u skladu sa CLSI EP smernicama. Ispitivanja su obuhvatala preciznost, linearnost, tačnost (trueness), granice detekcije i testiranje interferencija koriščenjem prikupljenih kliničkih uzoraka. Svi testovi su izvedeni na analizatoru LiCA® 800 (Chemclin, Beijing, Kina) sa reagensom iz serije 2202.

**Rezultati:**

Evaluacije proizvođača pokazale su izuzetne performanse testa: ponovljivost (1,41-4,39%), međukontrolna preciznost (1,62-7,29%) i reproduktivnost (1,62-7,22%). Metoda je pokazala linearnost u opsegu 0,10-16,64 ng/mL (ADL=10%). Upoređivanje metode sa MS referentnom metodom pokazalo je limit slaganja (LoA) od -19,5% do 14,4%, sa pristrasnošču &lt;10,00% na medicinskim tačkama odluke. Parametri senzitivnosti su uključivali: LoB (0,041 ng/mL), LoD (0,049 ng/mL) i LoQ (0,060 ng/mL pri 20% preciznosti). Test je pokazao visok stepen otpornosti na interferencije od strane hemoglobina (2 mg/mL), biotina (20 ng/mL), triglicerida (3 mg/mL) i bilirubina (0,157 mg/mL). Sve tvrdnje proizvođača potvrđene su nezavisnim kliničkim testiranjem od strane krajnjih korisnika.

**Zaključak:**

LiCA-T test zadovoljava sve kriterijume performansi definisane u najnovijim CLSI EP smernicama (uključujući EP06-Ed2 i EP07-Ed3), pokazujući pouzdanu analitičku preciznost i tačnost u svim ključnim parametrima validacije za kliničko određivanje testosterona.

## Introduction

Recent updates to Clinical and Laboratory Standards Institute (CLSI) guidelines, particularly EP07-Ed3 (Interference Testing in Clinical Chemistry, 3rd Edition) and EP06-Ed2 (Evaluation of Linearity of Quantitative Measurement Procedures, 2nd Edition) [1][2], have introduced significant methodological improvements over their predecessors. EP07-Ed3 supersedes EP07-A2 [3] by implementing new requirements for sample replicates and interferent volumes in paired-difference testing, while introducing a novel »point-to-point« approach for dose-response analysis. Similarly, EP06-Ed2 provides enhanced guidance on serum sample preparation, concentration selection, and introduces revised statistical methodologies compared to the previous EP06-A standard [4]. Although several studies have compared different editions of EP06 [5][6][7], practical applications of these updated protocols in product performance evaluation remain limited, and their general applicability continues to be debated in the scientific community [8][9][10][11].

Testosterone (T), a critical steroid hormone primarily secreted by gonadal tissues, serves dual physiological roles as both an estrogen precursor and a key regulator of follicular development in females [12]. Beyond its reproductive functions, emerging evidence links testosterone levels to various metabolic and cardiovascular pathologies, including atherosclerosis [13], coronary artery disease severity [14][15], type II diabetes [16], metabolic syndrome [17][18], and obesity [19]. These established associations underscore the clinical importance of accurate testosterone measurement in disease assessment and management.

The light-initiated chemiluminescent assay (LiCA) represents a significant advancement in homogeneous immunoassay technology, incorporating nanoparticle polymers, chemiluminescent signalling, and wash-free techniques. Previous validation studies have demonstrated the analytical robustness of LiCA platforms in measuring various hormones, including 17-estradiol [5], thyroid-stimulating hormone [20], and anti-Müllerian hormone [21], with excellent precision profiles.

Recently, Chemclin Diagnostics Co., Ltd. has developed a LiCA-based testosterone assay. However, comprehensive performance evaluations of this novel platform remain limited in the literature. In accordance with clinical laboratory accreditation requirements, we conducted a rigorous multi-parameter validation following current CLSI guidelines (EP05-A3 [22], EP15-A3 [23], EP06-Ed2 [2], EP09-Ed3c [24], EP17-A2 [25] and EP07-Ed3 [1]). This study systematically evaluates the analytical performance of LiCA for testosterone measurement across critical parameters, including precision, linearity, accuracy, detection capability, and interference resistance.

## Materials and methods

### Testosterone measurement

All serum samples were collected between March 10th and June 10th, 2023, and stored at -20°C until analysis. Serum testosterone (T) levels were quantified using the LiCA® 800 automated chemiluminescence immunoassay analyser (Chemclin, Beijing, China), along with the manufacturer’s matched reagents (Lot. 2202), calibrators, and quality control materials.

### Performance evaluation

### Imprecision

The manufacturer assessed imprecision following the CLSI EP05-A3 guidelines [22] using a multisite precision evaluation scheme. The study employed four samples – two human serum samples (near clinical decision levels, noted as Sample 1 and Sample 2) and two quality control materials (noted as QCL and QCH) – analysed via nested components of variance. Over five consecutive days, each sample was tested five times per day across three independent instrument systems. Data were used to calculate repeatability, intermediate imprecision, reproducibility, and their respective confidence intervals.

Imprecision was further verified by the end-user following CLSI EP15-A3 guidelines [23]. The protocol was adapted from the manufacturer’s approach, employing two serum samples (near clinical decision levels) and two quality control materials. Each sample was analysed in five replicates per day for five consecutive days on a single instrument system. Potential outliers were detected using the Grubbs test, and precision estimates (repeatability and intermediate imprecision) were derived through one-way analysis of variance (ANOVA) and subsequently validated.

### Linearity interval

The manufacturer evaluated the linearity interval according to CLSI EP06-Ed2 guidelines [2]. Eleven linearity panels were prepared with concentrations spanning from the lower limit of quantitation (LLOQ) to the upper limit of quantitation (ULOQ). The required number of test replicates was determined based on repeatability performance and allowable deviation from linearity (ADL, set at 10.00%). Measurement results were analysed using weighted least squares (WLS) regression to obtain predicted values for each concentration level. Linearity interval was assessed by comparing the relative deviation between predicted values and measured means against the predefined ADL criteria.

Linearity interval verification was conducted following CLSI EP06-Ed2 [2] using high- and low-concentration samples within the manufacturer-specified linear range (LLLI to ULLI). Each sample was analysed in quadruplicate. Potential outliers were identified through scatter plot analysis. The WLS regression model was applied to evaluate the mean values and dilution proportions (PH, i). The calculated linearity deviations and their confidence intervals were then compared against the ADL requirements for verification.

### Trueness/accuracy

Trueness was evaluated following CLSI EP09-A3 guidelines [24] using isotope dilution-liquid chromatography-tandem mass spectrometry (ID-LC-MS/MS) as the reference method. The measurement system was calibrated with certified reference materials from the Australian National Metrology Institute: NMIA-M914b (testosterone) as the primary standard and NMIA-D546 (d3-testosterone) as the internal standard.

The validation protocol included repeated testing of serum panels with reference method-assigned values, comparative analysis of two independent datasets, outlier exclusion using established statistical criteria, bias estimation through Bland-Altman analysis (mean relative deviation and limits of agreement) and Passing-Bablok regression (medical decision point evaluation). Trueness was considered verified when: (1) all calculated bias estimates met predefined quality specifications, (2) the regression-derived bias at medical decision levels was within acceptable limits, and (3) Bland-Altman agreement parameters satisfied validation criteria.

### Detection capability

Detection capability was assessed according to the EP17-A2 [25] guidelines. The following parameters were included: limit of blank (LoB), limit of detection (LoD), and limit of quantitation (LoQ). LoB was estimated using the non-parametric statistical approach by testing samples with a measurand concentration of 0. LoD was estimated by testing 5–7 samples, among which the lowest and highest concentrations were close to LoB and 4 times LoB, respectively. If the variance homogeneity test revealed homogeneous results (*p*>0.05), a classical approach was used to calculate the LoD. Otherwise, the imprecision profile approach was used. Then, the power-function model (Origin 2019b software) was applied to calculate LoQ by testing a series of samples which cover the lower limit of the measurement interval. The allowable imprecision level was set to 6.98%.

The clinical end-users also evaluated the detection capability, according to the EP17-A2 guidelines. The performance was considered verified when the following items were met: (1) LoB, 87% of the test results were less than or equal to the LoB; (2) LoD, 87% of the test results were greater than or equal to the LoB; (3) LoQ, more than 90% of the test results were within the error margin (target value × [1 ± total error]).

### Interference

The interference was estimated for bilirubin, haemoglobin, biotin and triglycerides, according to the EP07-Ed3 [1] guidelines. The corresponding concentrations of interferences were based on the WS/T416-2013 guidelines (the »Guidelines for Interference Experiments« issued by the Chinese Health Administration). Furthermore, paired-difference experiments were carried out. If the concentration of interferences recommended by the guideline is determined to be interfering during the paired-difference experiments, the dose-response relationship was then estimated to determine the concentration of each interference substance. Samples containing interferents were mixed in a series of proportions with control samples to obtain samples containing interferents of different concentrations. Point-to-point curve fitting and linear regression were performed using a series of samples d_obs_ (X) and interferents concentrations (Y). As for the verification, merely the paired-difference experiment was conducted by the clinical end user. During the interference evaluation and verification, d_max_ (maximum allowable difference) was predefined as 15% of the sample concentration.

### Statistical analysis

Nested components of variance and WLS regression analysis were performed using SPSS 25.0, while the Grubbs and ESD outlier test, Bland-Altman analysis, and Passing-Bablok regression model were conducted using MedCalc 13.3.3. Other analyses, such as one-way ANOVA, scatter plot for the percentage of deviation from predicted values, linearity analysis plot, imprecision curve plotting, and »point-to-poin« curve fitting, were performed using MS Excel. The power curves were generated using the Origin 2019b software. A *p*-value of <0.05 was considered statistically significant.

## Results

### Estimates performed by the manufacturer

### Imprecision

Statistical analysis using the Grubbs test confirmed the absence of outliers in all datasets. The mean of QCL and QCH for imprecision estimate was 1.75 ng/mL and 4.36 ng/mL, which fall in the range of the manufacturer’s requirement. The means of Sample 1 and Sample 2 were calculated to be 3.38 ng/mL and 7.11 ng/mL, which were near the upper limit of the reference interval. The assay demonstrated excellent precision performance across all evaluation parameters, with coefficients of variation (CVs) being as follows: repeatability (within-run precision) for QCL, QCH, Sample 1 and Sample 2: 3.61%, 3.55%, 3.24%, 1.67% respectively; intermediate imprecision and reproducibility (total precision): 4.84%, 4.40%, 4.56%, 2.14% respectively.

### Linearity

The linearity evaluation was performed using serial dilutions (based on a proportion of 1:9, 2:8, 3:7…9:1) of high-concentration (16.64 ng/mL) and low-concentration (0.10 ng/mL) testosterone samples. The concentrations of eleven linearity panels were calculated to be the expected concentrations. Each dilution series was prepared in quadruplicate through proportional mixing, with all measurements demonstrating acceptable reproducibility (Grubbs test confirmed no outliers). Weighted least squares (WLS) regression analysis revealed excellent agreement between measured (X) and expected (Y) concentrations, yielding the regression equation: Y=0.999X (R^2^=0.999). The deviation from linearity (DL) of eleven linearity panels between measured concentrations and predicted concentrations which obtained through WLS regression equation ranged from -1.56% to 6.21%, within the ADL of ±10%, which demonstrated linear performance across the entire measurement range of 0.10-16.64 ng/mL with all data points falling within the predefined acceptance criteria.

### Accuracy

**Figure 1:**
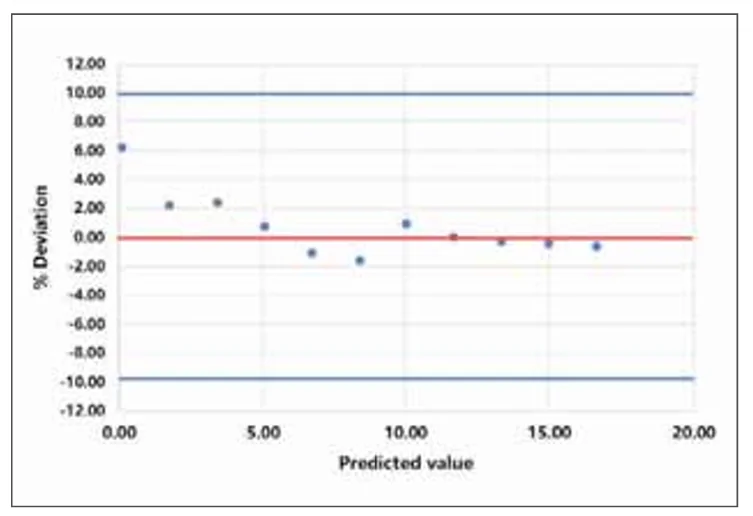
Scatter plot for the linearity interval assessment: percentage DL (Y axis) and predicted value (X axis).

The analytical accuracy of low-concentration testosterone measurements (<0.6 ng/mL) was evaluated using seven clinical samples spanning 0.17–0.53 ng/mL. Method performance was assessed against the manufacturer’s (Chemclin Diagnostics Co., Ltd.) specifications and the stringent quality standards established by the Royal College of Pathologists of Australasia Quality Assurance Programs (RCPAQAP), with an acceptance criterion of ±0.12 ng/mL for allowable bias. All test results demonstrated satisfactory agreement within the predefined tolerance limits (the bias from ID-LC-MS/MS results ranged from -0.06 to 0.07 ng/mL), thereby validating the assay’s accuracy for low-concentration testosterone quantification. Figure 1

For the samples (≥0.60 ng/mL), the analysis was carried out according to EP09-A3 guidelines.

### Outlier

The Extreme Studentized Deviate (ESD) method (α=0.01) identified no outliers for per cent bias between the measurement concentrations of LiCA 800 and reference concentrations obtained by ID-LC-MS/MS method across all 38 sample groups, which spanned a clinically relevant concentration range of 0.96–16.06 ng/mL.

### Bias estimation

**Figure 2:**
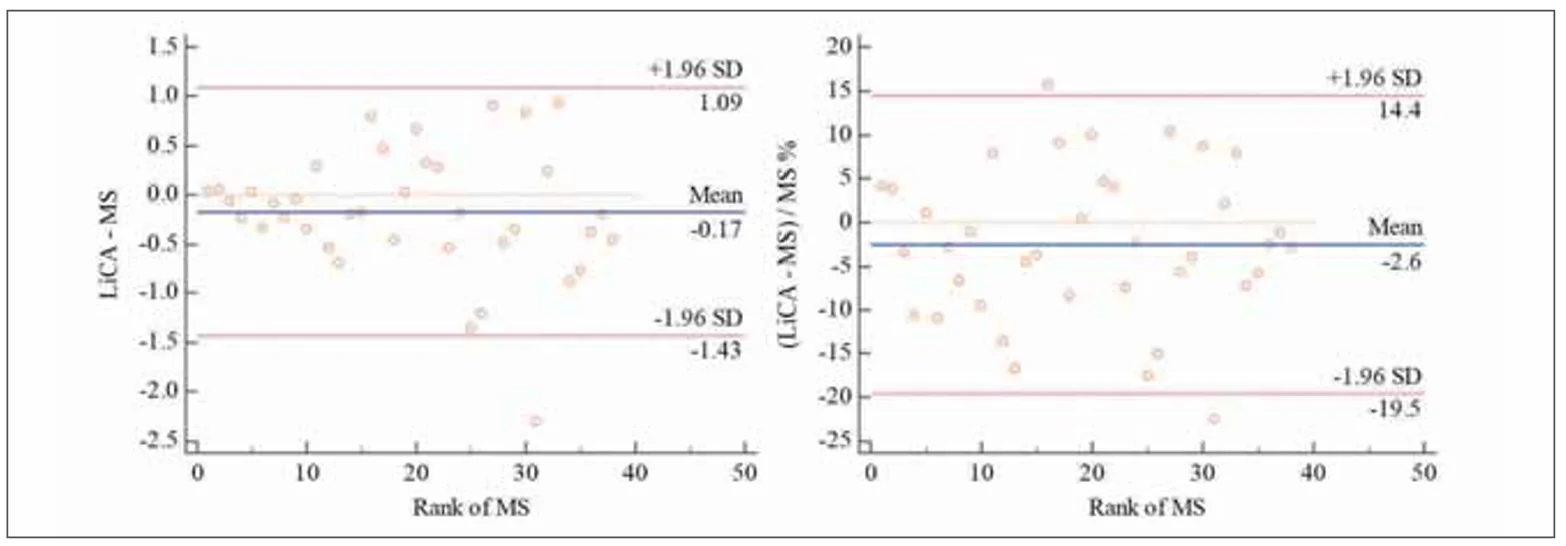
The Bland-Altman plots of bias and per cent bias by the manufacturer.

Bland-Altman analysis (Figure 2) demonstrated stable variation characteristics, with coefficient of variation (CV) values remaining consistent across the measurement range. Normality assessment via the Shapiro-Wilk test confirmed acceptable distribution of relative bias (W=0.9884, p=0.9574). The overall mean bias was -2.55% (95% confidence interval: -5.40% to 0.30%).

### Regression analysis

**Figure 3:**
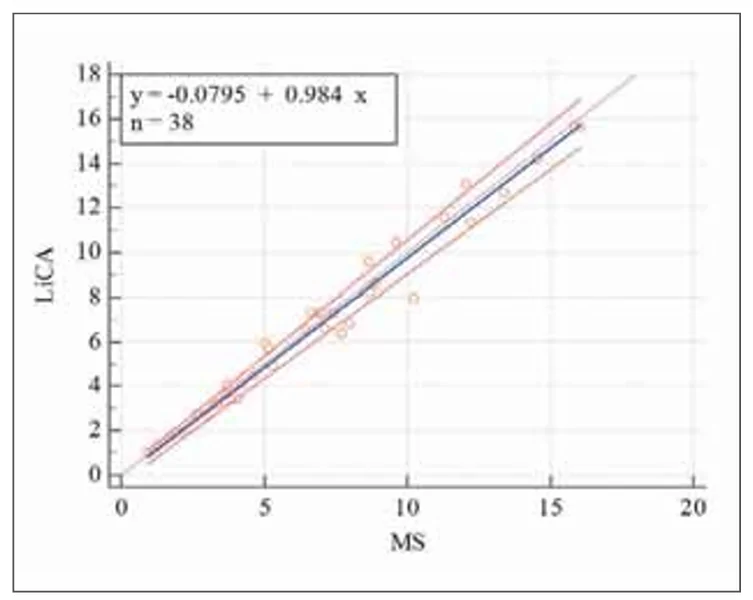
The Passing-Bablok regression curve estimated by the manufacturer.

For method comparison analysis, we employed Passing-Bablok regression due to its non-parametric nature and robustness against distributional assumptions. This approach yielded the regression equation Y=0.984X - 0.080 (95% CI for slope: 0.939–1.043; intercept: -0.370–0.121), demonstrating excellent agreement between methods. The strong correlation was further supported by Spearman’s rank correlation coefficient (ρ=0.981, p<0.001), as illustrated in Figure 3 and detailed in Table 1.

**Table 1:** Summary of the four linear fitting models. OLR, ordinary least squares; WLS, weighted least squares; CI, confidence interval.

Regression models	Regression curves	Slope (95% CI)	Intercept (95% CI)	Proportional bias (%)
OLR	Y=0.976X-0.011	0.924–1.029	-0.430–0.409	-7.6–2.9
WLS	Y=0.967X+0.033	0.923–1.011	-0.112–0.179	-7.7–1.1
Deming	Y=0.989X-0.097	0.949–1.029	-0.322–0.128	-5.1–2.9
Passing-Bablok	Y=0.984X-0.080	0.939–1.043	-0.370–0.121	-6.1–4.3

### Bias at the medical decision level

At critical medical decision concentrations (1.00, 3.00, and 7.50 ng/mL), calculated relative bias ranged from -10.00% to -2.68% based on the Passing-Bablok regression. All values fell within clinically acceptable limits.

### Detection capability

### Limit of blank

Using a non-parametric approach, we analysed 60 blank measurements (α=0.05) to determine the 95th percentile (PctB=0.95). The 95th percentile position was calculated as 57.5 [0.5+(60×0.95)]. The LoB was subsequently derived using the formula: LoB=X_57_ + 0.5(X_58_-X_57_, where X_57_=0.040 ng/mL and X =0.041 ng/mL. This yielded a final LoB of 0.041 ng/mL, indicating excellent assay specificity.

### Limit of detection

**Figure 4:**
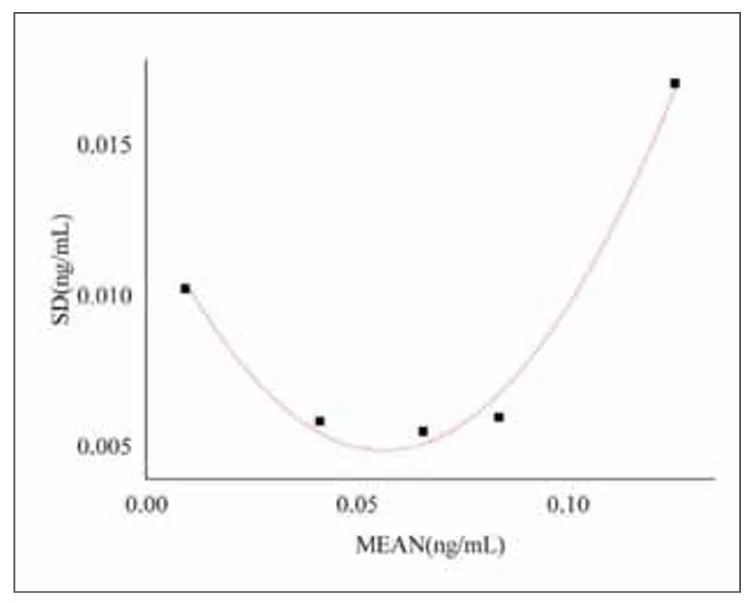
The estimated precision curve by the manufacturer: Y-axis, laboratory variation, X-axis, mean concentration.

Analysis of 25 replicate measurements revealed significant heteroscedasticity (Levene’s test: F=9.338, p<0.001), necessitating an imprecision curve approach. The quadratic imprecision function was established as:

SD = 2.5138(concentration)^2^- 0.2833(concentration) + 0.0129. Figure 4

By iteratively solving for the concentration where the measured signal exceeded the LoB by 1.645SD, we determined the LoD to be 0.049 ng/mL. This was achieved through a stepwise refinement process until measurement bias approached zero.

### Limit of quantitation

Through a 5-day precision study (11 samples, 5 replicates/day), we modelled the relationship between concentration and imprecision using power regression:

CV (%) = 2.26(concentration)^ (-1.21)

The assay demonstrated distinct quantitation capabilities at different precision thresholds: 0.215 ng/mL at 6.98% CV (stringent criteria); 0.060 ng/mL at 20.00% CV (standard clinical threshold). This multi-tiered characterisation provides laboratories with flexible implementation options based on their required precision standards.

### Interference

Following WS/T416-2013 guidelines, we evaluated potential interferents using two testosterone concentrations (3.14 ng/mL [high] and 0.57 ng/mL [low]). The paired-difference test was conducted with five replicates per sample, with interference defined as d_obs_ (the »point estimate« of the interference effect, is calculated as the difference between the mean measurand values of the test and control samples) exceeding d_max_ (±0.471 and±0.085 ng/mL for high and low samples, respectively).

**Figure 5:**
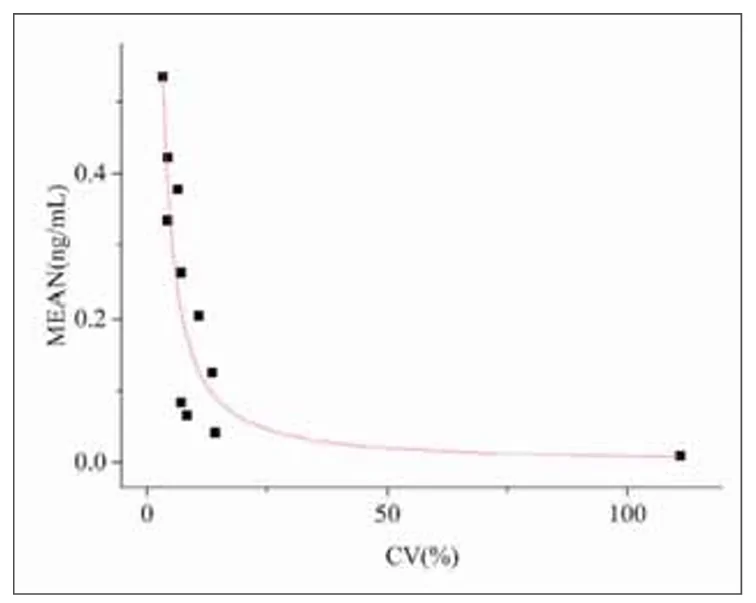
The power function curve estimated by the manufacturer.

Preliminary testing demonstrated no significant interference from: haemoglobin (≤2 mg/mL), biotin (≤20 ng/mL), triglycerides (≤3 mg/mL). The calculated d_obs_ for haemoglobin were 0.408 and 0.076 ng/mL for high and low samples, respectively, -0.180 and -0.028 mL for biotin, and -0.072 and -0.026 mL for triglycerides. However, bilirubin at 0.2 mg/mL showed clinically significant interference, where d_obs_ were 0.752 and 0.208 ng/mL for high and low samples, respectively. Figure 5

**Figure 6:**
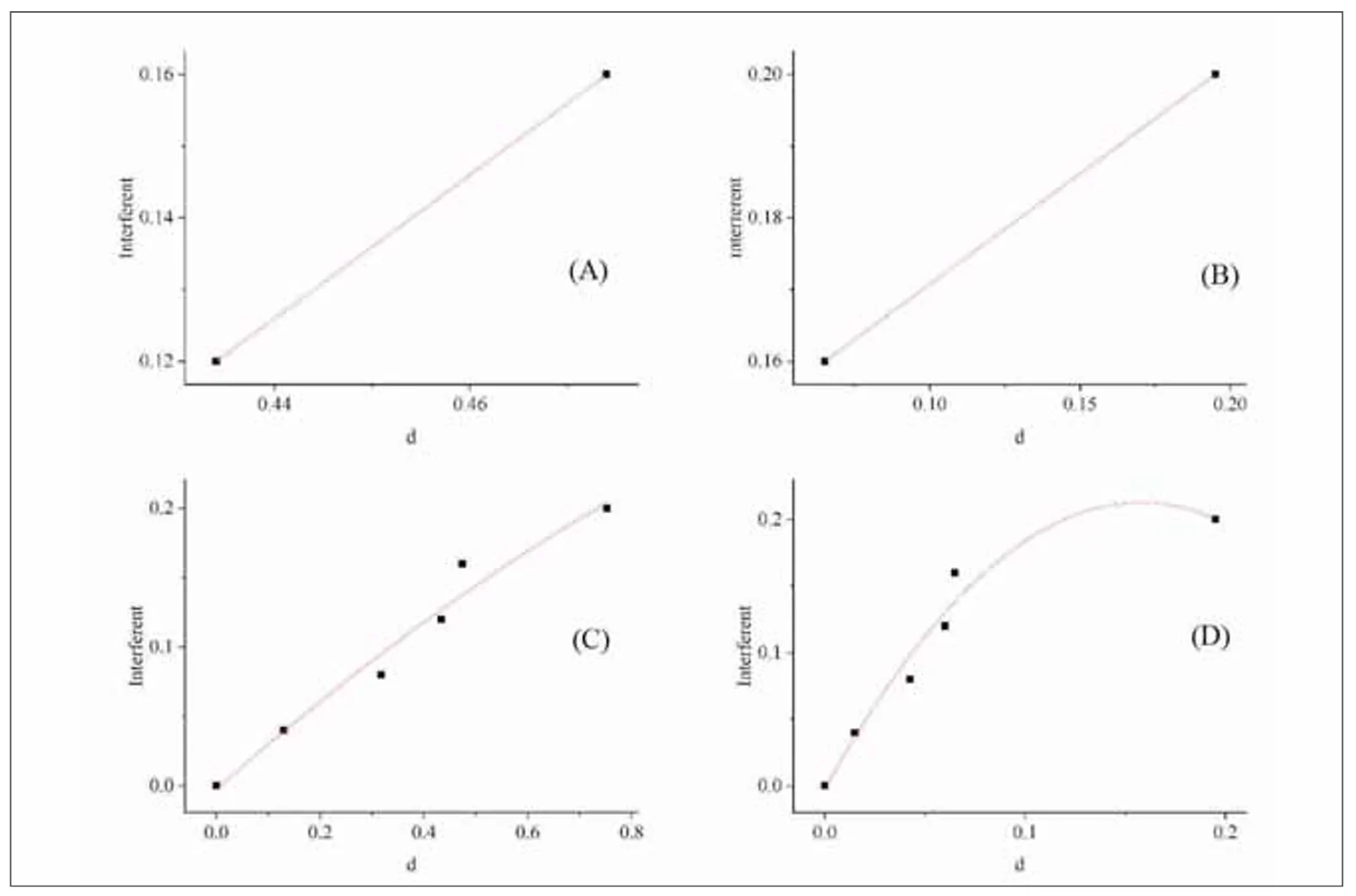
The interference results for the dose-response curve with different methods (by manufacturer).

Samples containing 0.2 mg/mL bilirubin were mixed with control samples to obtain samples containing bilirubin of different concentrations. Point-to-point curve fitting and linear regression were performed using a series of samples’ d_obs_ (X) and bilirubin concentrations (Y). The non-interference threshold for bilirubin obtained by fitting is 0.157 and 0.166 mg/mL for high and low samples, respectively, from the point-to-point curve (Figure 6A–Figure 6B), and 0.137 and 0.167 mg/mL from linear regression (Figure 6C–Figure 6D).

Using the more conservative point-to-point method according to EP07-Ed3, we established the following non-interfering thresholds: bilirubin (<0.157 mg/mL), haemoglobin (<2.00 mg/mL), biotin (<20.00 ng/mL), and triglyceride (<3.00 mg/mL). These results demonstrate the assay’s robustness against common interferents at clinically relevant concentrations.

### Performance verification by clinical end-users

### Imprecision verification

The repeatability and intermediate imprecision were calculated for each sample. The means of imprecision verification samples are 1.77, 4.58, 2.30, and 7.92 ng/mL for QCL, QCH, Sample1, and Sample2. Repeatability of the 4 samples is 3.68%, 2.44%, 2.09%, and 0.28%, and intermediate imprecision is 4.25%, 3.12%, 6.20%, and 1.01%, respectively. The results were consistent with the manufacturer’s declared performance specifications, thereby verifying their claims.

### Linearity verification

**Figure 7:**
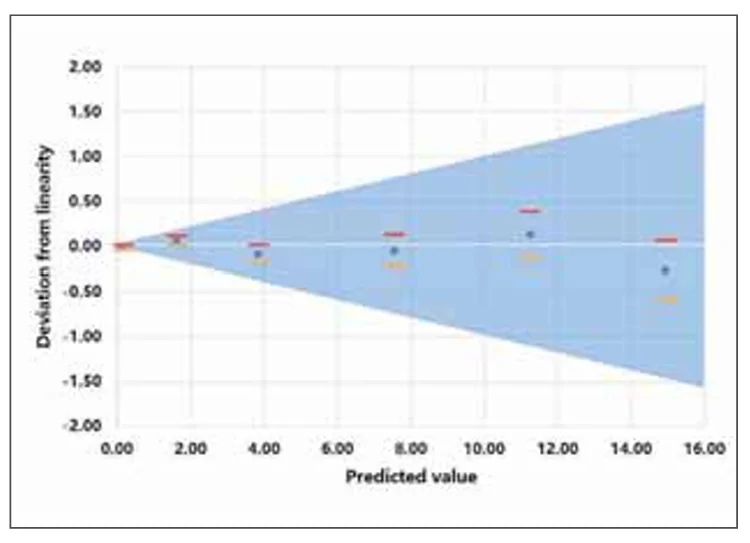
The linearity diagram constructed by the clinical end user.

Linearity was assessed using two samples (0.13 and 14.88 ng/mL), which were mixed into 6 samples. Each mixed sample was analysed in quadruplicate, and a regression equation was derived: Y = 14.795X + 0.133 (Y: measured mean value; X: dilution ratio), through which we can obtain the predicted concentration for each mixed sample. The DL, which was obtained from predicted concentration along with measured mean value and its confidence interval (CI), was determined and marked in Figure 7. Since all measurements fell within the ADL ±10%, the linearity interval was confirmed.

### Verification of detection capability

The manufacturer-reported limits of blank (LoB, 0.041 ng/mL) and detection (LoD, 0.049 ng/mL) were evaluated. All blank sample results where the measured concentrations ranged from 0.001 to 0.030 ng/mL were below the LoB, while all low-level samples where the measured concentrations ranged from 0.045 to 0.051 ng/mL exceeded the LoD, confirming the manufacturer’s claims.

For the limit of quantification (LoQ, 0.060 ng/mL), the manufacturer specified an allowable imprecision of 6.98%. Five clinical samples were assessed against this criterion, with 60 test results analysed. Although five measurements exceeded the error margin, 91.7% of results remained within the acceptable range, supporting the validity of the declared LoQ.

### Interference test

Potential interference from bilirubin (0.157 mg/mL), haemoglobin (2 mg/mL), biotin (20 ng/mL), and triglycerides (3 mg/mL) was evaluated using high- (5.39 ng/mL) and low-level (0.62 ng/mL) samples (five replicates each). The d_max_ was calculated to be ±0.809 and ±0.093 ng/mL for high and low samples, respectively. The calculated d_obs_ for bilirubin were -0.678 and 0.048 ng/mL for high and low samples, respectively, -0.344 and 0.054 ng/mL for haemoglobin, -0.198 and -0.016 ng/mL for haemoglobin, and -0.780 and 0.084 ng/mL for triglycerides. No significant interference was observed, confirming the manufacturer’s claims.

## Discussion

The LiCA® assay offers a simple, rapid, and high-throughput method for T quantification, with automation compatibility due to its wash-free workflow. In this study, the analytical performance of the LiCA® 800 system for T measurement was rigorously evaluated and verified in accordance with the latest CLSI EP guidelines.

Our results demonstrate that the LiCA® system reliably quantifies serum T concentrations with excellent precision, sufficient linearity interval, appropriate detection capability and satisfactory results in anti-interference characteristics. These findings confirm that the LiCA® T assay meets clinical performance standards, and its manufacturer-declared specifications were successfully verified by an independent clinical end user following CLSI EP guidelines. This underscores the robustness of the CLSI EP framework for both manufacturer validation and end-user verification.

The EP06-Ed2 [2] guideline introduces a simplified statistical approach using weighted linear regression when measurement repeatability is concentration-dependent, reducing the disproportionate influence of high-concentration samples. Unlike the EP06-A [4] method (which relies on polynomial fitting and comparison with linearity), EP06-Ed2 emphasises clinically acceptable deviations rather than strict statistical thresholds. Key improvements include Confidence interval (CI)-based evaluation (If the mean concentration of a measurement exceeds the ADL but its CI partially overlaps with the ADL, linearity can still be accepted) and Optimised sample selection (The guideline specifies appropriate high (H) and low (L) sample concentrations, adjusting for assay imprecision to ensure 95% of measurements yield valid results) [26][27].

The EP07-Ed3 [1] guideline recommends piecewise linear interpolation (“point-to-point” curve fitting) for interference assessment, offering greater flexibility than the polynomial fitting method in EP07-A2. Additional enhancements include: Increased repetitions (A minimum of five replicates per sample (vs. three in EP07-A2) [3] improves reliability) and Higher interferent stock concentration (At least 20× the test concentration (vs. 10× in EP07-A2) minimises matrix effects).

However, there were still some limitations and considerations while applying EP06-Ed2 and EP07-Ed3. EP06-Ed2 assumes a constant coefficient of variation (CV%) across the measuring interval, using unweighted regression with a forced zero intercept. If data violate this assumption, alternative weighting methods may be required, complicating analysis. EP07-A2 assumes local monotonicity for piecewise linear interpolation. Non-monotonic data may render this method inapplicable. These constraints highlight areas for potential refinement in future guideline iterations.

## Conclusion

The LiCA® assay for testosterone (T) demonstrates excellent analytical performance, fully meeting its specified performance criteria. This validation confirms that the LiCA® system provides precise and reliable measurement of serum T levels, making it well-suited for clinical applications. Combined with its rapid turnaround time and high-throughput capability, the LiCA® system emerges as a practical and efficient solution for T quantification in routine diagnostic workflows. Furthermore, the updated EP06 and EP07 guidelines offer a more rigorous and practical framework for performance evaluation, incorporating enhanced statistical methodologies to ensure robust assay validation.

## Dodatak

### Acknowledgements

We thank the National Center for Clinical Laboratories (NCCL, China), especially Prof. Tianjiao Zhang, for the traceability work that assigned our calibrator values.

### Research ethics

The study complies with all relevant national regulations, institutional policies, and the Helsinki Declaration (as revised in 2013), and was approved by the Ethics Committee of the Women & Infants Hospital of Zhengzhou.

### Informed consent

The patient’s consent was waived due to the anonymity of the serum samples used after the routine examination and the anonymity of the analysed data.

### Authors’ contributions

LA: conception and design; LA: administrative support; LA and XJC: provision of study materials or patients; XXR and XJC: collection and assembly of data; XXR and XJC: data analysis and interpretation. All authors wrote and approved the manuscript.

### Funding

None.

### Data availability 

The datasets generated during and/or analysed in the study are available from the corresponding author upon reasonable request.

### Conflict of interest statement

All the authors declare that they have no conflict of interest in this work.
